# Amlexanox, a selective inhibitor of IKBKE, generates anti-tumoral effects by disrupting the Hippo pathway in human glioblastoma cell lines

**DOI:** 10.1038/cddis.2017.396

**Published:** 2017-08-31

**Authors:** Yang Liu, Jie Lu, Zhimeng Zhang, Lin Zhu, Shicai Dong, Gaochao Guo, Ruohong Li, Yang Nan, Kai Yu, Yue Zhong, Qiang Huang

**Affiliations:** 1 Department of Neurosurgery, Tianjin Medical University General Hospital, Tianjin, China; 2 Key Laboratory of Post-Trauma Neuro-Repair and Regeneration in Central Nervous System, Ministry of Education, Tianjin, China; 3 Tianjin Key Laboratory of Injuries, Variations and Regeneration of Nervous System, Tianjin, China; 4 Department of Neurosurgery, Shandong Provincial Qianfoshan Hospital, Shandong, China; 5 Department of Pathology, The Second People’s Hospital of Liaocheng, Linqing, China

## Abstract

Glioblastoma multiforme (GBM) is the most prevalent form of malignant brain tumor. Amlexanox, a novel compound, has been shown to have anti-cancer potential. In this study, the anti-tumoral effects and the underlying mechanisms of amlexanox were investigated. Amlexanox significantly suppressed proliferation and invasion and induced apoptosis in glioblastoma cells. Furthermore, we found that amlexanox altered the protein expression of the Hippo pathway by downregulating IKBKE. Our data indicates that IKBKE directly targets LATS1/2 and induces degradation of LATS1/2, thereby inhibiting the activity of the Hippo pathway. *In vivo* results further confirmed the tumor inhibitory effect of amlexanox via the downregulation of IKBKE, and amlexanox induced no apparent toxicity. Collectively, our studies suggest that amlexanox is a promising therapeutic agent for the treatment of GBM.

GBM is the most common and most malignant tumor in the central neural system.^1^ The biological properties of GBM are strong proliferation, rapid migration, intensive invasion, genetic alteration and increased angiogenesis.^2^ Although there have been great advances in neuroimaging, neurosurgical techniques, radiotherapy and chemotherapy, the overall survival for patients with GBM has still remained unchanged for the last several decades.^3, 4^ The median survival time of GBM patients is currently 15–17 months from diagnosis, which is only a few months longer than 30 years ago. The 5-year overall survival rate of GBM is ~5.1%.^5, 6^ Recently, an increasing number of studies have been focusing on the identification of novel molecular targets that are crucial for developing more effective therapies.^7^ IKBKE (inhibitor of nuclear factor kappa-B kinase subunit epsilon, also called IKK*ε* or IKK-i), which is a member of the I*κ*B kinase (IKK) family, has been identified as an oncogenic protein and found to be upregulated in breast cancer,^8^ ovarian cancer,^9^ prostate cancer^10^ and glioma.^2, 11^ Over-expression of IKBKE inhibited glioma cell apoptosis and, in contrast, siRNA-mediated suppression of IKBKE increased the sensitivity of glioma cells to apoptotic inducers.^11^ Our previous study indicated that IKBKE is an emerging key regulator of the malignant progression of gliomas, which is associated with a poor prognosis, and silencing of IKBKE by siRNA can reduce the proliferative and invasive abilities of glioma cells, highlighting it as a potential therapeutic target.^2^

Amlexanox is an anti-inflammatory, anti-allergic, immunomodulator and has been developed for the treatment of ulcers, allergic rhinitis and asthma in the clinic.^12^ Recently, amlexanox was found to be an inhibitor of the protein kinases TBK1/IKBKE and improve obesity related metabolic dysfunction.^13^ In the present study, we identified amlexanox as a potent anti-glioma drug by downregulating IKBKE.

The Hippo pathway is an evolutionarily conserved regulator of tissue and organ growth and controls multiple cellular functions that are central to tumorigenesis, including proliferation and apoptosis.^14^ The Hippo pathway comprises the STE20-like protein kinase1 (MST1, also known as STK4) and MST2 (also known as STK3), large tumor suppressors 1 and 2 (LATS1/2), MOB kinase activator (MOB1A/B), SAV1 and neurofibromatosis 2 (NF2). When the Hippo pathway is activated, MST1/2 phosphorylate and activate LATS1/2 with the help of SAV1. Then, LATS1/2 phosphorylate and restrict the activity of two transcriptional co-activators, Yes-associated protein (YAP) and transcriptional coactivator with a PDZ-binding motif (TAZ).^15, 16^ The Hippo pathway regulates the nuclear translocation of YAP1, which is an important downstream player, and has been found to have a possible link with stem/progenitor cells, organ size and cancer.^17^ YAP1 has been implicated as an oncoprotein and may promote glioblastoma growth, and high levels of expression has been associated with clinically aggressive glioblastoma subtypes.^18^ We investigated the mechanism in which IKBKE may function as a negative regulator of the Hippo pathway through controlling LATS1/2 stability and YAP1 activity.

In this study, we demonstrated the effects of amlexanox on the migration and invasion of glioblastoma cells through the downregulation of IKBKE levels, which ultimately targets the Hippo pathway. Amlexanox is a potentially attractive anti-neoplastic agent that might benefit therapy for GBM in the future.

## Results

### Amlexanox suppressed GBM cells proliferation and induced G0/G1 phase arrest

**Figure 1:**
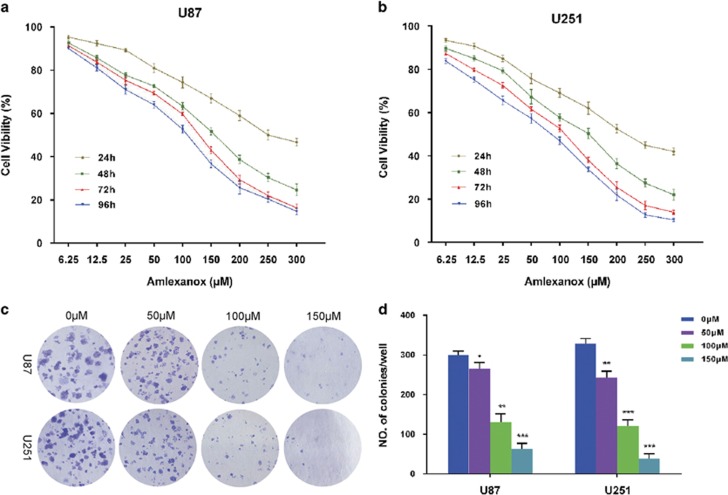
Amlexanox inhibited proliferation and colony formation of glioma cells. (**a** and **b**) U87 and U251 cells were treated with amlexanox (0–300 *μ*M) for 24, 48, 72 and 96 h, and cell vibility was detected using CCK-8 assay. (**c**) U87 and U251 cells were treated with various concentrations of amlexanox (0, 50,100 and 150 *μ*M) for 2 weeks. (**d**) The cell colonies were stained and quantified, and the results are presented in the histogram. The results shown are representative of at least three independent experiments. Data are shown as the mean±S.D. *P<0.05 and **P<0.01 compared with the control group

To investigate the effects of amlexanox on the proliferation of glioma cells *in vitro*, the viability rate of U87 and U251 was assessed by CCK-8. Supplementary Figure 1 shows amlexanox chemical structure. Treatments of amlexanox inhibited cell viability in a dose- and time-dependent manner at different concentrations (6.25, 12.5, 25, 50, 100, 150, 200, 250 and 300 *μ*M) for 24–96 h compared with control cells (Figures 1a and b). The IC50 values for 72 h of amlexanox treatment were approximately 140 *μ*M in U87 and 120 *μ*M in U251 cells. Based on the IC50 measured in the study, concentrations of 50, 100 and 150 *μ*M were selected to be used in the following experiments. Furthermore, the ability of both glioma cell lines to form colonies was evaluated with or without amlexanox treatment for a period of 2 weeks (Figure 1c). The results showed that colony formation rates were significantly reduced in the amlexanox-treated groups in both glioma cell lines (Figure 1d).

**Figure 2:**
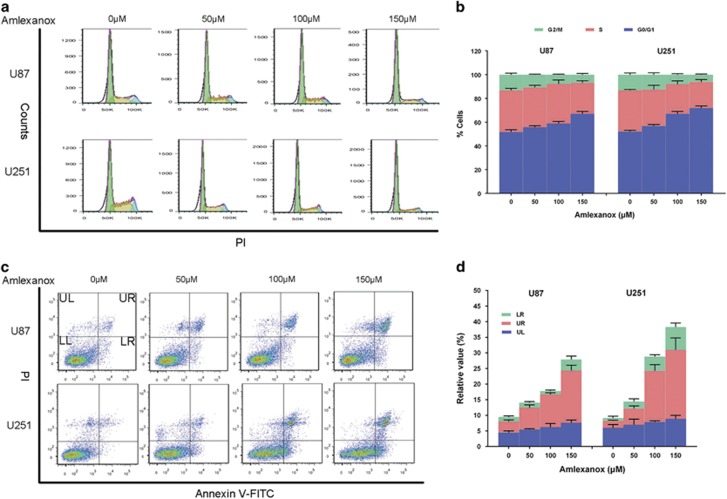
Amlexanox induced G0/G1 phase arrest and apoptosis in glioma cells. (**a**) U87 and U251 cells were treated with various concentrations of amlexanox for 72 h and then, assessed by propidium iodide staining-coupled flow cytometry. (**b**) Bar graphs showing a concentration-dependent cell cycle arresting effect of amlexanox in U87 and U251 cells. (**c**) After treatment with various concentrations of amlexanox for 72 h, U87 and U251 cells were stained with annexin V-FITC and PI for analysis using flow cytometry. (**d**) Increased apoptotic cells in U87 and U251 were quantified and presented in the histogram. Data are shown as the mean±S.D. *n*=3 for each group. *P<0.05 and **P<0.01 compared with the control group

The effect of amlexanox on the cell cycle progression of glioma cells was evaluated by DNA content analysis. U87 and U251 cells were treated with various concentration of amlexanox for 72 h. Amlexanox induced cell cycle arrest in a concentration-dependent manner (Figures 2a and b). Treatment with 150 *μ*M amlexanox caused a significant increase in G0/G1 and decrease in the S and G2/M populations of glioma cells, compared with that control. These results suggested that amlexanox inhibited the transition from G0/G1 to S phase, leading to G0/G1 arrest, which was related to the inhibition of cells proliferation.

### Amlexanox induced glioma cell apoptosis

Annexin V/PI analysis was used to assess the apoptosis of U87 and U251 cells after various concentrations of amlexanox treatment for 72 h. As shown in Figures 2c and d, the percentages of apoptotic cells increased in a concentration-dependent manner. After 150 *μ*M amlexanox treatment in U87 cells, the percentage of early apoptotic cells was elevated from 1.47 to 3.58%, and the percentage of late apoptotic cells was elevated from 3.17 to 16.7%, whereas in U251 cells the percentage of early apoptotic and late apoptotic cells increased from 3.17 and 2.23% to 16.7 and 22.13%, respectively.

### Amlexanox attenuated the migration and invasion of glioma cells

**Figure 3:**
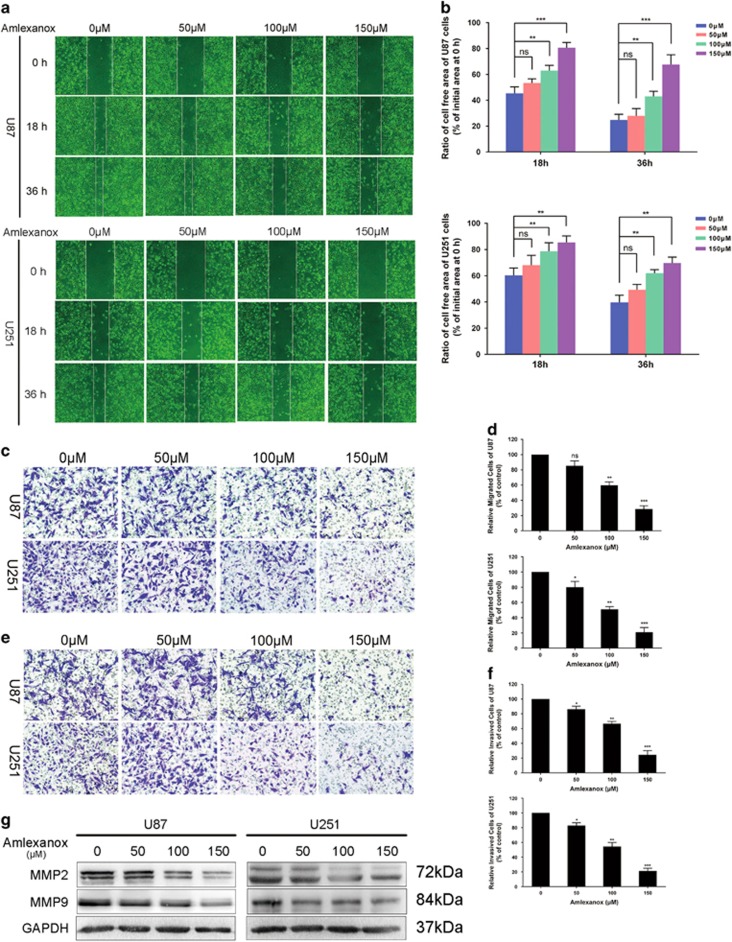
Amlexanox inhibited migration and invasion of glioma cells. U87 and U251 cells were treated with amlexanox at various concentrations of 0, 50, 100 and 150 *μ*M for 72 h. (**a**) Representative images from the wound healing assay of each cell are displayed for 18 or 36 h after cell seeding. (**b**) The results of the wound healing were quantified and presented in the histogram. (**c**) Representative images of the transwell migration assay (without Matrigel) are shown for 12 h after U87 cells seeding and 24 h after U251 cells seeding. (**d**) Statistical analysis of migrating cell numbers. (**e**) Representative images of the transwell invasion assay (with Matrigel) are shown for 24 h after U87 cells seeding and 48 h after U251 cells seeding. (**f**) Statistical analysis of migrating cell numbers. (**g**) The protein levels of MMP-2 and MMP-9 were evaluated by western blot. The results shown are representative of at least three independent experiments. Data are shown as the means±S.D. *P<0.05 and **P<0.01 compared with the control group

The effects of amlexanox on migration and invasion of U87 and U251 cells were examined by the wound healing assay and transwell assay. The results of the wound healing assay showed that healing decreased gradually with increased concentrations of amlexanox (Figures 3a and b). Amlexanox at 150 *μ*M significantly reduced the migration capability of U87 and U251 cells compared with that of control. The results of the wound healing assay were also supported by that of the transwell migration assay (Figures 3c and d).

Amlexanox impaired the invasion of U87 and U251 cells across transwell chambers compared with that observed in control (Figures 3e and f). Matrix metalloproteinases (MMPs), such as MMP-2 and MMP-9, play important roles in the invasion and malignancy of glioma cells.^19^ After U87 and U251 cells were treated with amlexanox at various concentrations for 72 h, the protein expression levels of MMP-2 and MMP-9 were checked by western blot (Figure 3g). The results showed that amlexanox inhibited the expression of MMP-2 and MMP-9 in a dose-dependent manner, and 150 *μ*M amlexanox significantly inhibited the expression of MMP-2 and MMP-9 compared with that in control.

### Amlexanox reverted the malignant phenotype of GBM cells via activating the Hippo pathway mediated by downregulation of IKBKE

**Figure 4:**
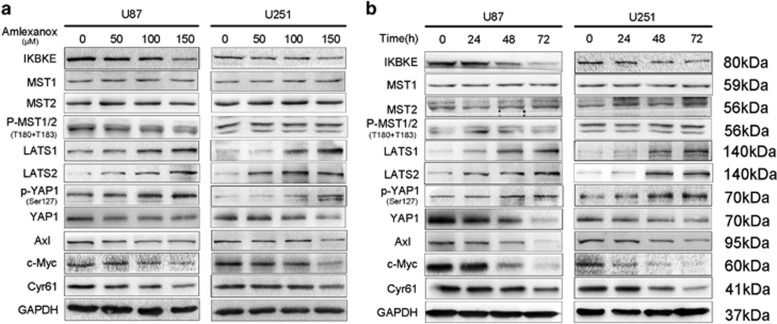
Amlexanox reduced the protein expression of the Hippo pathway through downregulation of IKBKE. (**a**) U87 and U251 cells were treated with various concentrations of amlexanox for 72 h. The cells were harvested, and the effects of amlexanox on the protein expression of the Hippo pathway were detected by western blot. (**b**) U87 and U251 cells were treated with amlexanox at 150 *μ*M for 24, 48 and 72 h. Cell lysates were collected, and the protein expression were detected by western blot as in (**a**)

Amlexanox has been shown to block IKBKE activity by interacting with the enzyme in the ATP-binding region, and amlexanox treatment reduced IKBKE protein levels in brown adipose tissue and non-small cell lung cancer cells.^13, 20^ Since we have observed the inhibitory effect of amlexanox on glioma cells, we next investigated the effect of amlexanox on molecular signals in the U87 and U251 cell lines, including the Hippo pathway. The Hippo pathway controls tissue growth and cell fate, whereas pathway deregulation can induce tumors in model organisms and occurs in a broad range of human carcinomas.^14^ We found that amlexanox treatment could inhibit the IKBKE protein and alter the abundance and phosphorylation status of the Hippo pathway proteins in glioma cells. As shown in Figures 4a and b, first, we examined the level of IKBKE using western blot and found that there was a significant decrease in IKBKE expression in response to amlexanox treatment. However, the qRT-PCR results showed that the IKBKE mRNA level remained unchanged after amlexanox treatment, suggesting that amlexanox did not affect IKBKE transcription (Supplementary Figures 2a–d).

Compared with the levels in the control cells, amlexanox treatment increased the level of LATS1/2 and phospho-YAP1 (Ser127), whereas YAP1 and the downstream targeted effectors of the Hippo pathway, including Axl, c-Myc and Cyr61,^21^ were significantly reduced in a dose-and time-dependent manner. In contrast, the levels of protein for MST1/2 and phospho-MST1/MST2 (Thr183 in MST1 and Thr180 in MST2) did not change after amlexanox treatment.

### IKBKE inactivates the Hippo pathway by downregulation of LATS1/2

**Figure 5:**
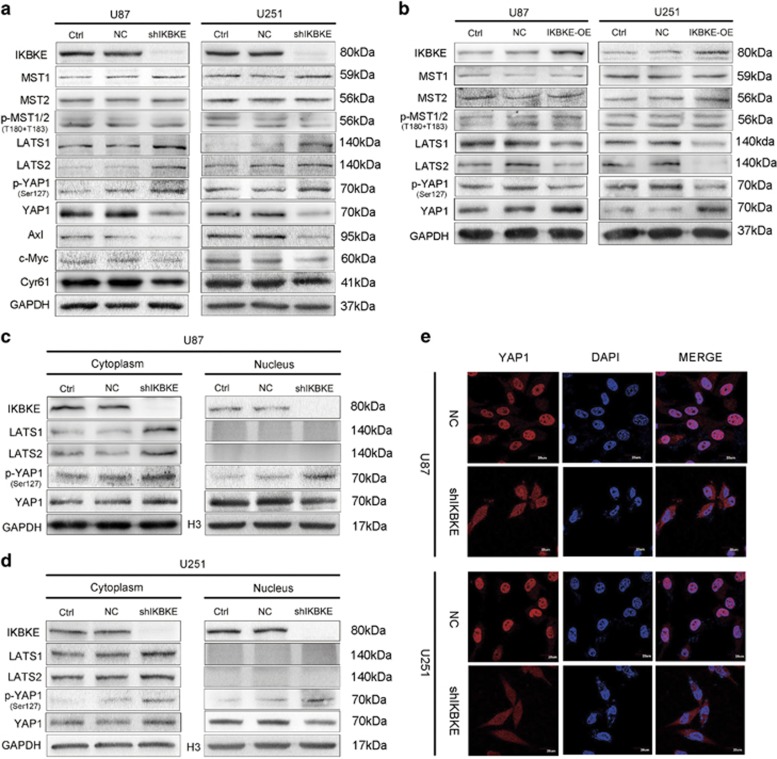
The Hippo pathway was involved in the proliferative regulation of glioma cells mediated by IKBKE. (**a**) U87 and U251 cells were transfected with control shRNA or IKBKE shRNA for 48 h, and the effects of IKBKE knockdown on the protein expression of the Hippo pathway were determined by western blot. (**b**) Glioma cells were transfected with IKBKE-expressing plasmid for 48 h, and the effects of IKBKE up-regulation on the protein expression were determined by western blot as in **a**. (**c** and **d**) Expression of YAP1 in the cytoplasmic and nuclear fractions was examined by western blot. (**e**) IKBKE-knockdown U87 and U251 cells were stained with DAPI and the antibody against YAP1 (scale bar=20 *μ*m)

Next, we identified the potential molecular mechanisms by which IKBKE affects the Hippo pathway. The results showed that IKBKE knockdown in U87 and U251 cells dramatically elevated LATS1/2 and phospho-YAP1 (Ser127) proteins and suppressed YAP1 protein and the downstream targets of the Hippo pathway, while nearly had no effect on the expression of MST1/2 and phospho-MST1/MST2 (Thr183 in MST1 and Thr180 in MST2) proteins (Figure 5a). In contrast, the over-expression of IKBKE in U87 and U251 cells decreased LATS1/2 and phospho-YAP1 (Ser127) protein expression and increased the expression of YAP1 proteins, whereas it similarly did not change the levels of MST1/2 and phospho-MST1/MST2 (Thr183 in MST1 and Thr180 in MST2) proteins (Figure 5b), suggesting the very possible involvement of the Hippo pathway in IKBKE-mediated glioma growth and that IKBKE could regulate LATS1/2 and YAP1 in the pathway.

As shown in Figures 5c and d, compared to the control, the downregulation of IKBKE triggered a reduction of YAP1 in the total and nuclear protein extracts of U87 and U251 cells, but not in the cytoplasm. In addition, the immunofluorescence analysis further confirmed the change in the cytoplasm and nucleus localization of YAP1 when IKBKE was silenced (Figure 5e; Supplementary Figures 3a and b and b). YAP1 phosphorylation on serine 127 is a direct target of LATS1/2 kinase.^22^ Phosphorylation of YAP1-Ser127 by LATS1/2 generates a 14-3-3 binding site and induces YAP1 cytoplasmic retention and degradation.^23, 24^

### IKBKE downregulates LATS1/2 by promoting their degradation

**Figure 6:**
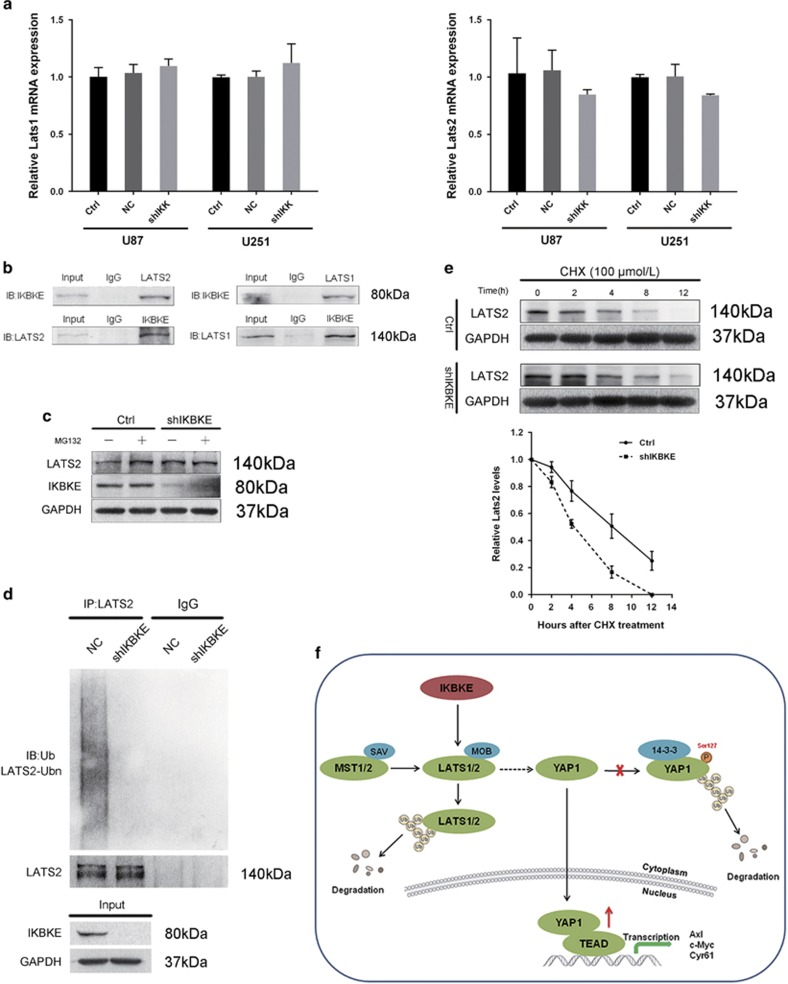
IKBKE interacts with and degrades LATS2. (**a**) Expression of LATS1/2 in IKBKE knockdown and control cells was examined at transcript levels. (**b**) The endogenous interaction between IKBKE and LATS1/2 in U87 cells was analyzed by immunoprecipitation. (**c**) LATS2 expression in IKBKE-knockdown U87 cells and control cells was measured after treatment with or without 20 *μ*M MG132 for 12 h. (**d**) Decreased LATS2 ubiquitylation level by IKBKE knockdown in U87 cells. (**e**) Measurement of LATS2 in cell lysates harvested at 0, 2, 4, 8 and 12 h after the addition of CHX (100 *μ*M) to arrest protein synthesis. (**f**) Schematic diagram of the mechanism of amlexanox-mediated antitumor activity by downregulation of IKBKE in GBM. IKBKE directly binds to and negatively regulates LATS1/2, which promotes YAP1 cytoplasmic retention and subsequent degradation

We further found that there was no significant difference between the control and shIKBKE groups for LATS1/2 mRNA expression, which was determined by qRT-PCR (Figure 6a), indicating that IKBKE may function as an endogenous regulator of LATS1/2 at the post-translational level.

To explore the interaction between IKBKE and LATS1/2, we performed Co-IP experiments with the IKBKE and LATS1/2 antibody. The result indicated the existence of combination between IKBKE and LATS1/2 in glioma cells (Figure 6b). It has been reported that LATS2 destabilization is mainly regulated by proteasome and ubiquitin pathway.^22, 25, 26^ After treatment with the proteasome inhibitor MG132, we observed that the IKBKE-induced reduction of LATS2 expression was blocked (Figure 6c). we next analyzed LATS2 ubiquitination levels in IKBKE knockdown U87 cells. IKBKE knockdown decreased LATS2 ubiquitination, indicating that IKBKE may regulate LATS2 stability through promoting its polyubiquitin degradation (Figure 6d). To measure the kinetics of LATS2 degradation, we used cycloheximide (CHX) to inhibit protein synthesis. The LATS2 protein degraded faster in the control cells than in the shIKBKE cells (Figure 6e). Since IKBKE interacted with both LATS1 and LATS2, which are homologous to each other,^27^ suggesting that IKBKE may be involved in the regulation of LATS1 through identical mechanisms.

Taken together, the results indicated that IKBKE may function as a negative regulator of the Hippo pathway through controlling LATS1/2 stability and YAP1 activity.

### Amlexanox suppresses glioblastoma growth in xenograft models

**Figure 7:**
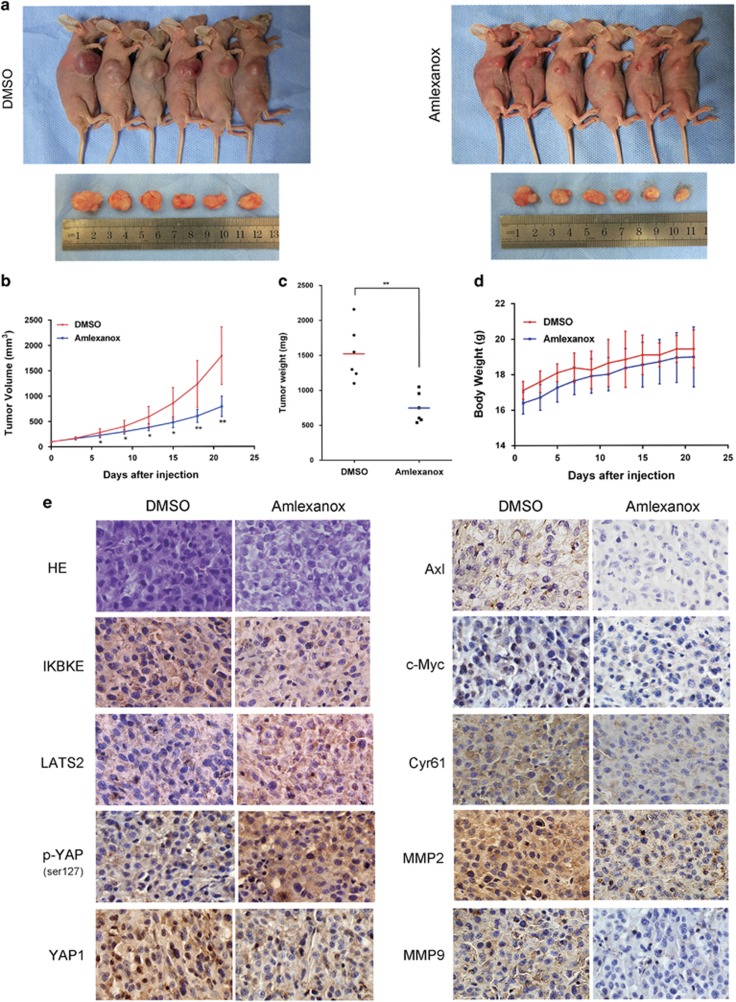
Amlexanox exhibited significant antitumor effects against subcutaneous tumors *in vivo*. (**a**) Images of nude mice and tumors from the DMSO and amlexanox treatment groups. (**b**) The tumor volumes and (**c**) weights, and (**d**) the body weights of mice were evaluated using the *in vivo* proliferation assay. (**e**) Representative images of the HE and immunohistochemical staining for IKBKE and the proteins of the Hippo pathway in tumor sections (× 200 magnification). IKBKE protein was effectively inhibited by amlexanox treatment. Besides, the expressions of LATS2 and p-YAP1(Ser127) increased, whereas YAP1, Axl, c-Myc, Cyr61, MMP-2 and MMP-9 expression levels were simultaneously decreased in amlexanox-treated group relative to the DMSO-treated group. Data are shown as the mean±S.D. *P<0.05, **P<0.01, compared to the control (*n*=6)

To investigate the potential effects of amlexanox on glioblastoma growth *in vivo*, we first established a subcutaneous glioma model using U87 cells as described previously. Seven days after implantation, DMSO or amlexanox were intraperitoneally injected every day for 21 days. Amlexanox treatment exhibited an inhibitory effect on subcutaneous tumor growth and significantly decreased the tumor volume compared with that observed in the control group on day 21 (Figures 7a and b). Moreover, the tumor weight of mice treated with amlexanox was reduced by 50.9% compared with that of the control group (Figure 7c). In addition, no significant differences in body weights were observed between amlexanox treatment and the control group (Figure 7d). Amlexanox did not alter organs morphology nor induce hemorrhage in the heart, lung, kidney, spleen and liver (Supplementary Figures 4a and b).

**Figure 8:**
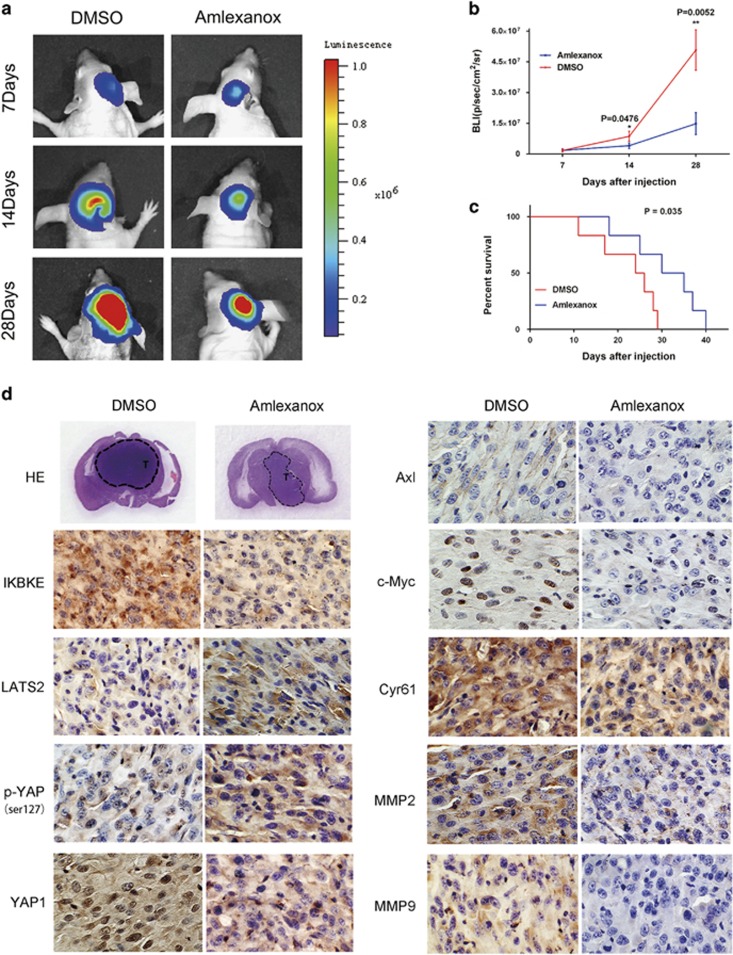
Antitumor effects of amlexanox in a U87 orthotopic intracranial model. The mice were treated with intraperitoneal injection with DMSO or amlexanox (100 mg/kg) daily. The treatment started from the 7th day after implantation and lasted for ~21 days. (**a**) Representative images of bioluminescence of mice on days 7, 14, and 28 after implantation. (**b**) Quantitative analysis of these bioluminescence images for the DMSO and amlexanox treatment groups. (**c**) The overall survival of mice in the DMSO and amlexanox treatment groups. There was a substantial survival benefit for the amlexanox-treated mice. (**d**) Representative images of the HE and immunohistochemical staining for IKBKE and the Hippo pathway proteins in tumor sections (× 200 magnification). IKBKE protein was effectively inhibited by amlexanox treatment. Besides, the expressions of LATS2 and p-YAP1(Ser127) increased, whereas YAP1, Axl, c-Myc, Cyr61, MMP-2 and MMP-9 expression levels were simultaneously decreased in amlexanox-treated group relative to the DMSO-treated group. Data are shown as the mean±S.D. *P<0.05, **P<0.01 compared to the control (*n*=6)

To further confirm the therapeutic potential of amlexanox, orthotopic intracranial mouse models were constructed using the U87 glioma cell line, followed by amlexanox or DMSO treatment. The results of the bioluminescence imaging (BLI) showed that tumor growth was delayed in the amlexanox treatment group compared to that in the DMSO group (Figures 8a and b). Amlexanox-treated mice survived markedly longer than DMSO-treated mice. The results suggested that amlexanox could prolong the survival time of tumor-bearing mice (Figure 8c). As shown in Figure 8d, IHC showed that the expression levels of IKBKE was significantly lower in the brain tumor tissue of amlexanox-treated mice. The results suggested that amlexanox had an effect on glioma cells *in vivo* by downregulating IKBKE. Moreover, the expression of LATS2 and p-YAP1(Ser127) increased, whereas YAP1, Axl, c-Myc, Cyr61, MMP-2 and MMP-9 expression levels were simultaneously decreased in amlexanox-treated group relative to the DMSO-treated group, which was consistent with the subcutaneous tumor tissues results (Figure 7e).

## Discussion

IKBKE, which is a member of the IKK family, is known as a non-canonical IKK. Previous studies have illustrated that IKBKE plays a key role in inflammatory and metabolic diseases.^12, 13, 28, 29, 30, 31, 32^ Recently, IKBKE has been implicated as an oncogenic protein and found to be upregulated/activated in multiple cancers. Over-expressing IKBKE can result in malignant transformation in various cell types.^8^ Thus, IKBKE has been considered as a promising therapeutic target for the treatment of cancer.

As a small molecular inhibitor of IKBKE, previous reports have illustrated that amlexanox has anti-inflammatory, anti-allergic, immunomodulation activity, and it is used for treatment of aphthous ulcer, asthma, allergic rhinitis and obesity in the clinic.^12, 33^ In the present report, we showed that amlexanox displays anti-glioma properties *in vitro* with weak adverse effects on normal cells. Amlexanox is cytotoxic to glioma cells with IC50 values in the micromolar concentrations. Incubation of glioma cells with amlexanox inhibits cellular proliferation, migration and invasion and induces G0/G1 phase arrest and apoptosis in U87 and U251 cells. In addition, amlexanox capable of directly binding to IKBKE and modulating its activity, and a reducing the protein levels of IKBKE have been reported.^13, 20^ In contrast, the mRNA level of IKBKE was not decreased after amlexanox treatment, suggesting that amlexanox does not affect IKBKE transcription. Therefore, further studies are needed to demonstrate the mechanism by which amlexanox treatment induces downregulation of IKBKE protein levels.

Many previous studies have focused on downstream targets of IKBKE. IKBKE has been reported to activate the NF-*κ*B pathway by interacting with several cellular proteins,^34, 35, 36^ that induce expression of the pro-tumorigenic cytokines IL-6 and CCL5 and subsequent activation of the JAK/STAT pathway.^37^ In addition, IKBKE can activate AKT by directly phosphorylating AKT on Thr308 and Ser473 independent of mTORC2.^38^ IKBKE is also able to inhibit FOXO3a by inducing FOXO3a nuclear-cytoplasmic translocation and protein degradation.^39^

The Hippo pathway can suppress cell proliferation and invasion, promote apoptosis and control organ size in diverse species, whereas pathway deregulation can induce tumorigenesis in model organisms and occurs in different kinds of human tumors, including lung, ovarian, colorectal and liver cancer.^14^ LATS1/2 could regulate cell migration through YAP and YAP-regulated transcriptional activity.^40^ YAP1 is a downstream transcriptional activator of the Hippo pathway, and is phosphorylated and inactivated by the pathway cascades. The loss of the Hippo pathway or aberrant activation of YAP1 will lead to nuclear accumulation of YAP1, thus promoting the expression of genes required for cell proliferation through direct binding to their cognate transcription factors.^41^

As a serine/threonine protein kinase, we speculate that IKBKE possibly participates in the regulation of the Hippo pathway, which is a kinase cascade and activation of LATS1/2 kinases (and inactivation of YAP/TAZ) represents the major functional output of the pathway. To confirm that amlexanox inhibited cell growth through the Hippo pathway activated by downregulation of IKBKE, we examined the protein levels of the Hippo pathway after amlexanox treatment. The results of showed that amlexanox could regulate LATS1/2 and YAP1 phosphorylation in a dose- and time-dependent manner. However, the protein levels of MST1/2 and the phosphor-levels of MST1/2 (Thr183 in MST1 and Thr180 in MST2), which could phosphorylate and activate LATS1/2, were not affected. Thus, we believe that the Hippo pathway might be one of the most important downstream pathways for the antitumor activity of amlexanox against GBM. To fully understand the possible mechanism by which IKBKE affects the Hippo pathway, we demonstrated that knockdown or over-expression of IKBKE affected the abundance of LATS1/2, phosphorylation status of YAP1 and the expression of Axl, c-Myc and Cyr61, which are the downstream targets of YAP1 by interaction with TEAs.^21^ However, we did not detect a significant change in MST1/2 and phospho-levels of MST1/2 (Thr183 in MST1 and Thr180 in MST2) upon IKBKE over-expression or depletion. Furthermore, we demonstrated, for the first time, that IKBKE directly bound to LATS1/2, and facilitated their polyubiquitin degradation. Meanwhile, our data showed that IKBKE did not alter mRNA levels of LATS1/2 in glioma cells. All of above data support the conclusion that IKBKE regulates the Hippo pathway through post-translational control of LATS1/2.

Amlexanox also exhibited promising antitumor efficacy in subcutaneous glioma xenograft models, which is consistent with its strong anti-proliferative activity *in vitro*. To verify whether amlexanox is able to cross the blood brain barrier (BBB), orthotopic intracranial mouse models were constructed. The data demonstrated that amlexanox not only significantly reduced brain tumor growth and the expression of IKBKE but also prolonged the survival of the intracranial models, suggesting a good BBB permeability of amlexanox.

In conclusion, our study is the first to demonstrate that amlexanox displays promising anticancer activity against glioma cells harboring high levels of IKBKE *in vitro* and *in vivo*. We further demonstrate a new role for IKBKE as a negative regulator of LATS1/2 and an inactivator of the Hippo pathway. However, it should be noted out that the effects on other IKBKE-regulated pathways may also contribute to the activity of amlexanox in glioma cells. These findings indicate that amlexanox is a promising chemotherapeutic compound for the treatment of GBM.

## Materials and methods

### Cell lines and materials

Human glioma cell lines (U87 and U251) were purchased from the American Type Culture Collection (ATCC) (Manassas, VA, USA). The cells were cultured in Dulbecco’s Modified Eagle’s Medium (DMEM) (Gibco, USA) supplemented with 10% fetal bovine serum (FBS) (Gibco) and were incubated in a humidified atmosphere of 5% CO_2_ at 37 °C. Amlexanox (HY-B0713) was purchased from MedChem Express (MCE) (Shanghai, China), and a 150 mM stock solution was prepared in dimethyl sulfoxide (DMSO) (Sigma Chemical, St. Louis, MO, USA). Antibodies against LATS1/2, MMP-2, MMP-9 and Cyr61 were purchased from Abcam (Shanghai, China). Antibodies against IKBKE, YAP1, p-YAP1 (Ser127), c-Myc and Ubiquitin were purchased from Cell Signaling Technology (Beverly, MA, USA). The Axl antibody was purchased from Bioss (Beijing, China).

### CCK-8 cell viability assay

Cell viability was measured by a Cell Counting Kit-8 (CCK-8) assay (Dojindo, Japan). U87 and U251 cells (4 × 10^3^ cells per well) were planted into 96-well plates and incubated for 12 h. Then, the cells were treated with different concentrations of amlexanox (0–300 *μ*M). After incubation with amlexanox for 24, 48, 72 or 96 h, CCK-8 was added to each well, and cells were incubated for 1.5 h. The optical density was measured at 450 nm (OD_450_) using the microplate reader (Synergy2, BioTech, Vermont, VT, USA). Three independent experiments were performed.

### Cell colony formation assay

U87 and U251 cells were seeded in six-well plates at a density of 2000 cells per well. After 12 h incubation, cultured cells were treated with various concentrations of amlexanox (50, 100 and 150 *μ*M). The culture medium was changed once every three days. The cells were grown for an additional 14 days. The cell colonies were fixed with methanol and stained with crystal violet. Colonies (⩾50 cells) were counted under an inverted microscope (Olympus, Japan). The rate of colony formation in each group was analyzed. Each experiment was performed in triplicate.

### Cell cycle analysis

Glioma cells were seeded in six-well plates (1 × 10^5^ cells per well). The cells were treated with DMSO or amlexanox for 72 h and then, harvested by trypsinization without EDTA. The cells were fixed with ice-cold 70% ethanol and incubated at 4 °C overnight. Then, the cells were stained for total DNA content with RNase (BD Biosciences, USA) and propidium iodide (PI, BD Biosciences). The samples were detected using a FACScan flow cytometer (BD Biosciences), and cell cycle was analyzed using FlowJo software V 7.6.

### Cell apoptosis analysis

As described above, glioma cells were treated with DMSO or amlexanox. Then, the cells were stained with Annexin V-FITC and PI according to the manufacturer’s instructions of the Annexin V-FITC Apoptosis Detection kit (KeyGEN BioTECH, Nanjing, China). Cell apoptosis was analyzed using a FACScan flow cytometer (BD Biosciences), and the data were analyzed using FlowJo software.

### Scratch wound healing assay

U87 and U251 cells were pre-treated with amlexanox for 72 h. Then, the cells were seeded in six-well plates (5 × 10^5^ cells per well) until they reached 80–90% confluence. The scratch wounds were created in the monolayer of confluent glioma cells with a 200 *μ*l pipette tip. Then, these cells were incubated at 37 °C and in 5% CO_2_. Wound healing was measured and recorded photographically over time using a phase-contrast microscope at 0, 18 and 36 h.

### *In vitro* migration assay

The migratory capacity of glioma cells was evaluated using transwell filters (8 *μ*m pore size, Corning Costar, NY, USA). U87 and U251 cells were pre-treated with amlexanox for 72 h. The cells were resuspended in serum-free DMEM at a density of 25 × 10^5^ per ml, and 200 *μ*l of glioma cell suspension was added into the upper chambers. Then, 500 *μ*l of DMEM supplemented with 10% FBS was placed in the lower chambers. After 12 h or 24 h of incubation, the cells that had migrated to the underside surface were fixed with paraformaldehyde and stained with crystal violet. The average number of migratory cells was counted under an inverted microscope (Olympus Corp., Japan) over five random fields in each well. All experiments were performed in triplicate.

### *In vitro* invasion assay

The effects of amlexanox on the invasion of glioma cells were elevated using a transwell invasion assay with inserts (8*μ*m pore size). The membranes of the transwell inserts were coated with Matrigel (BD Bioscience) and diluted with serum-free DMEM at a ratio of 1:3. U87 and U251 cells were prepared as described above. After 24 h or 48 h of incubation, the inserts were taken out and prepared for observation under an inverted microscope as described above. All tests were performed in triplicate.

### Construction of the lentiviral vector system, transfection and immunofluorescence staining

A lentivirus-mediated short hairpin RNA (shRNA) was designed to knockdown the expression of IKBKE in glioma cells. The target sequence of the IKBKE shRNA was 5′-GCA TCA TCG AAC GGC TAA ATA-3′, and a scrambled shRNA sequence of 5′-TTC TCC GAA CGT GTC ACG TTTC-3′ served as a negative control. These shRNAs were ligated into the pFH-L vector (GeneChem, Shanghai, China) containing green fluorescent protein (GFP). The constructed lentiviral vectors were transfected into U87 and U251 cells according to the manufacturer’s instructions. Successful infection was observed under a fluorescence microscope by the presence of green fluorescence in cells due to GFP. For immunofluorescence analysis, U87 and U251 cells were transfected with lentiviral vectors to detect the distribution of IKBKE, and YAP1 in the cytoplasm and nucleus, and the images were captured with an FV-500 laser-scanning confocal microscope (Olympus, Japan).

### Overexpression of IKBKE

The IKBKE and control vector plasmids were obtained from Addgene (Cambridge, MA, USA). The plasmids were transfected into U87 and U251 cells by using Lipofectamine 3000 (Invitrogen, USA) according to the manufacturer’s protocol.

### Western blot analysis, co-immunoprecipitation assays and ubiquitination assay

Glioma cells were treated with amlexanox (50, 100 and 150 *μ*M) for 72 h or treated with 150 *μ*M amlexanox for 24, 48 and 72 h. Cultured cells were lysed in RIPA buffer supplemented with protease inhibitor. The protein concentrations were evaluated with a BCA protein assay kit (Pierce, USA). The samples were heated in 100 °C water for 10 min to denature the proteins, separated on 10 or 12% SDS-PAGE gels, and electrophoretically transferred to a PVDF membrane (Millipore, USA). For the co-immunoprecipitation (Co-IP) experiments, glioma cells were lysed in IP Lysis/Wash Buffer (Thermo, USA). Fifty microliters of protein G agarose (Santa Cruz Biotechnology, CA, USA) was mixed with a specific monoclonal antibody or a nonspecific IgG overnight at 4 °C. The immunoprecipitated proteins were separated by SDS-PAGE and analyzed by western blot. For endogenous LATS2 ubiquitination assay, Lysates were incubated using 2 *μ*g anti-LATS2 antibody and analyzed by western blot using anti-Ubiquitin or anti-LATS2 antibodies.

### qRT-PCR analyses

Total RNA was prepared from glioma cells by using the Trizol reagent (Invitrogen) according to the manufacturer’s instructions. Reverse transcription was performed using the GoScript Reverse Transcriptase kit (Promega, Madison, WI, USA). qRT-PCR was carried out as follows: 10 min at 95 °C followed by 40 cycles of 15 s at 95 °C and 40 s at 55 °C. The primer sequences used for qRT-PCR are shown in Supplementary Table 1.

### Nude mouse model

To evaluate whether amlexanox inhibits tumor growth *in vivo*, the U87 cell line was selected to establish a nude mouse model. Female BALB/c nude mice (3–4 weeks, 11–14 g) were purchased from the Animal Center of the Cancer Institute at Chinese Academy of Medical Science (Beijing, China). To establish subcutaneous model, 5 × 10^6^ U87 cells in 200 *μ*l serum-free DMEM were injected into the right hind flank region of each mouse. After the tumors reached a volume of 100 mm^3^, the nude mice were randomized into two groups (*n*=6 per group). Then, amlexanox (100 mg/kg/day) or DMSO was injected intraperitoneally into the mice everyday for three weeks. The tumor volume was measured every 3 days using a digital caliper and calculated as follows: volume(mm^3^)=length × width^2^/2. At the end of the experiment, all mice were anesthetized and sacrificed, and tumors were weighed. To establish an intracranial model, 5 × 10^4^ U87 cells with a luciferase-encoding lentivirus were injected into the mice (*n*=6 per group) stereotactically. Luciferase-encoding lentivirus were purchased from GeneChem (Shanghai, China). The lentivirus vector is GV260: Ubi-MCS-Luc-IRES-Puromycin. After 7 days post implantation, the mice were injected intraperitoneally with amlexanox (100 mg/kg/day) or DMSO everyday during the survival period. Intracranial tumor growth was detected by using BLI on days 7, 14 and 28, using the IVIS Spectrum Live Imaging System (PerkinElmer, Branford, USA). The animal research was performed according to the internationally recognized guidelines and national regulations. Subcutaneous tumors and brains were extracted and fixed in 10% formalin and then, embedded in paraffin for HE and IHC.

### Statistical analysis

All results were expressed as the mean±S.D. Statistical analysis was performed with SPSS 16 software. The differences between two groups were assessed with a Student’s *t*-tests and differences between multiple groups were assessed using a one-way analysis of variance (ANOVA) test followed by Tukey’s *post hoc* test. *P*<0.05 was considered to be statistically significant.
