# Supplementary material for: Amlexanox, a selective inhibitor of IKBKE, generates anti-tumoral effects by disrupting the Hippo pathway in human glioblastoma cell lines
Source: Cell Death Dis. 2017 Aug 31;8(8):e3022–. doi: 10.1038/cddis.2017.396 (PMC5596579; doi:10.1038/cddis.2017.396)
Supplement: Supplementary Table 1 [file cddis2017396x2.docx]

| **Gene** | **Forward primer** | **Reverse primer** |
| --- | --- | --- |
| **GAPDH** | 5’-GGAGCGAGATCCCTCCAAAA-3’ | 5’-GGCTGTTGTCATACTTCTCATGG-3’ |
| **IKBKE** | 5’-GAGAAGTTCGTCTCGGTCTATGG-3’ | 5’-TGCATGGTACAAGGTCACTCC-3’ |
| **LATS1** | 5’-AATTTGGGACGCATCATAAAGCC-3’ | 5’-TCGTCGAGGATCTTGGTAACTC-3’ |
| **LATS2** | 5’-ACCCCAAAGTTCGGACCTTAT-3’ | 5’-CATTTGCCGGTTCACTTCTG-3’ |

**Supplementary Table 1：The primer sequences used for qRT-PCR.**
