# Supplementary material for: Amlexanox, a selective inhibitor of IKBKE, generates anti-tumoral effects by disrupting the Hippo pathway in human glioblastoma cell lines
Source: Cell Death Dis. 2017 Aug 31;8(8):e3022–. doi: 10.1038/cddis.2017.396 (PMC5596579; doi:10.1038/cddis.2017.396)
Supplement: Supplementary Figure Legends [file cddis2017396x3.docx]

**Supplementary Figure 1. Structure of amlexanox**

**Supplementary Figure 2. The relative IKBKE mRNA levels were analyzed by qRT-PCR.**

(a) U87 or (b) U251 cells were treated with 150 μM of amlexanox for the indicated time periods (0-72 h).

(c) U87 or (d) U251 cells were treated with various concentrations of amlexanox for 72 h. The cells were collected and analyzed by qRT-PCR (n = 3).

**Supplementary Figure 3.** **IKBKE expression levels and the subcellular location were confirmed using a confocal microscope.**

U87and U251 cells were transfected with IKBKE shRNA (GFP, green). After 48 h of transfection, the cells were stained with the IKBKE antibody (red) and DAPI (blue).

**Supplementary Figure 4. Amlexanox showed low toxicity in vivo.**

After treatment of amlexanox for 21 days, representative images of organs (heart, lung, liver, spleen and kidney) removed from DMSO and amlexanox (100mg/kg) treated nude mice.
